# Plant Behavioral Ecology

**DOI:** 10.3390/plants14152407

**Published:** 2025-08-04

**Authors:** Pavol Prokop

**Affiliations:** 1 Department of Environmental Ecology and Landscape Management, Faculty of Natural Sciences, Comenius University, Ilkovičova 6, 842 15 Bratislava, Slovakia; pavol.prokop@savba.sk; 2 Institute of Zoology, Slovak Academy of Sciences, Dúbravská Cesta 9, 845 06 Bratislava, Slovakia

The beginning of plant behavioral ecology cannot be easily defined because plant behavioral research has been separated from animals for a long time. The foundation of behavioral ecology could be rooted in earlier works of Niko Tinbergen [1], Geoff Parker [2], William Hamilton [3,4], Robert Trivers [5,6,7], John R. Krebs and Nicholas B. Davies [8], Edward O. Wilson [9], and others. Although behavioral responses to environmental stimuli, such as nyctinasty [daily leaf movements] and heliotropism [movement towards the sun], were well known centuries ago [albeit interpreted as purely mechanical rather than sensitive responses], a framework for plant behavior was developed relatively recently [10] and remains a target of criticism [11]. Darwin’s conceptualization of the “root-brain hypothesis” [12], where the root tip was compared to a brain responding to environmental cues, revolutionized the traditional view of plants as passive organisms to active responders [13]. Unfortunately, behavioral ecologists have traditionally built their principles of behavior exclusively using animals as models, whereas behavioral research on plants has remained dispersed for a long time in plant ecology and plant physiology journals. Therefore, it is my pleasure to see papers focused on plant behavioral ecology featured in this Special Issue of *Plants*, contributing to our understanding of plant behavior in a manner similar to previous efforts in the field, such as the Special Issue introduced by Cahill [14].

The Special Issue on plant behavioral ecology is represented by five papers that solve intriguing but conceptually different topics. Two papers were dedicated to the interactions between plants and ants. Pimenta and her co-workers [15] experimentally induced herbivory in a savanna plant, *Banisteriopsis malifolia* (Malpighiaceae), and found exciting interconnections between plant reproductive success and ant foraging. More intense foliar damage resulted in an increase in extrafloral nectar sugar concentration and an increased presence of aggressive plants defending damaged plants. Moreover, intense herbivory decreased plant reproductive success in terms of delayed blooming, number of inflorescences per plant, and decreased fruit size. In line with modern reasoning about the active role of plants in their environment, their results suggest that plants actively allocate resources (sugar in extrafloral nectaries) and strategically and sensitively respond to biological stress produced by predators.

Dos Santos and his colleagues [16] similarly showed that simulated foliage damage of *Eriotheca gracilipes* (Malvaceae) resulted in increased ant visitation which consequently decreased herbivory rates. Interestingly, the authors observed a significant shift from increased activity of extrafloral nectaries in young leaves, which are more vulnerable to herbivory, to increased foliar toughness in adult leaves, that is, a transition from indirect to direct defence against herbivores. Importantly, more intense leaf damage resulted in greater asymmetry of newly flushed leaves, supporting the idea that environmental stress shapes phenotypic development.

Al-Bakre [17] investigated the ecological factors underlying the climbing behavior of *Ochradenus baccatus* Delile (Resedaceae), a common desert plant in Saudi Arabia. This plant can be found growing independently as well as a facultative climber on the surrounding vegetation. Surveys conducted at 103 sites between 2020 and 2024 revealed that climbing individuals were associated with greater tree and shrub cover, higher elevation, and moderate soil fertility. In contrast, independent individuals were predominantly distributed in herbaceous and open habitats. The study on circumnutation, originally described by Darwin [12] and further investigated in *Pisum sativum* by Guerra et al. [18], demonstrates how plant apices and tendrils perform dynamic movements. Combined with evidence of *O. baccatus* strategically utilizing the surrounding vegetation, these findings reinforce the concept of plants as active participants in shaping their environment.

Finally, my colleagues and I [19] investigated why the early flowering plant *Ficaria verna* (Ranunculaceae) temporarily closes and reopens its flowers (nyctinasty). Two independent field experiments revealed that flowers experimentally prevented from closing retained fewer pollen grains and showed lower pollen viability than those that were experimentally prevented from closure. From a broader behavioral perspective, this study shows that flower opening and closure is not only a mechanistic response to environmental factors [e.g., rain], but it ultimately functions to enhance plant fitness.

**Concluding Remarks**

Five papers in this Special Issue represent new insights into three conceptually independent issues: plant defense, climbing behavior and circumnutation, and temporal flower closure. As the Guest Editor of this Special Issue, I sincerely thank all the contributing authors for their exceptional work, which significantly enhances our knowledge of the rapidly growing field of plant behavior. I am equally grateful to the reviewers for their time and thoughtful critiques and to the Editorial Office for their exceptional support throughout the publication process.
